# High-Frequency Ultrasonography—Possibilities and Perspectives of the Use of 20 MHz in Teledermatology

**DOI:** 10.3389/fmed.2021.619965

**Published:** 2021-02-22

**Authors:** Adriana Polańska, Dorota Jenerowicz, Elżbieta Paszyńska, Ryszard Żaba, Zygmunt Adamski, Aleksandra Dańczak-Pazdrowska

**Affiliations:** ^1^Department of Dermatology and Venereology, Poznań University of Medical Sciences, Poznań, Poland; ^2^Department of Dermatology, Poznań University of Medical Sciences, Poznań, Poland; ^3^Department of Integrated Dentistry, Poznań University of Medical Sciences, Poznań, Poland

**Keywords:** teledermatology, melanoma, atopic dermatitis, mycosis fungoides, high-frequency ultrasonography

## Abstract

High-frequency ultrasonography (HF-USG) is a non-invasive and *in vivo* method of visualization of the skin and upper part of subcutaneous tissue based on ultrasounds above 20 MHz. Although initially HF-USG was introduced to measure skin thickness, it currently gained widespread acceptance in dermato-oncology, primarily when used to determine skin tumor margins. Moreover, its application in different dermatology fields is known, particularly as a rapidly evolving method in the objective evaluation of the severity of various chronic skin diseases. Among different specialties, teledermatology belongs to leading and continually developing areas of successful telemedicine applications. Various skin conditions are visible to the human eye, which makes them particularly suitable for telemedicine. However, HF-USG enables specialists to look into deeper skin layers, thus extending diagnostic options. On the other hand, teledermatology creates the possibility of sending images for consultation and facilitates the therapeutic decision as HF-USG can be used in an asynchronous store and forward manner. It seems that HF-USG and teledermatology may be regarded as a truly matched pair. The aim of this work is to present current applications of 20-MHz ultrasonography in dermatology, including skin neoplasms and chronic skin diseases. Moreover, the authors aimed to analyze the possibilities of HF-USG use as a valuable tool in teledermatology, especially in diagnosing and monitoring patients suffering from long-lasting skin conditions.

## Introduction

According to the World Health Organization (WHO), telemedicine utilizes communication technologies in healthcare to exchange medical information for the diagnosis, treatment, prevention, research, evaluation, and education over a distance (1). Therefore, it can be considered a merge of expertise and communication technology, giving patients the possibility of being examined, monitored, and managed by a medical expert in a distant location. Electronic transfer of information may be accomplished in various ways. Still, two basic types currently in practice are “real-time” or “live interactive” (LI) (enables direct communication sender–recipient with an immediate result) and “store-and-forward” (SAF) telemedicine (digital images and patient data are captured, transferred, and stored) (2–4). Teledermatology belongs to the earliest and leading areas of the successful use of telemedicine solutions. Nowadays, it is applied among all kinds of medical facilities, including hospitals, primary care, or nursing homes (5, 6).

The principle aim of teledermatology seems to be to consult and to educate. Digital communication brings possibilities for the exchange of medical information between the patient and the specialist. What is more, a recent modality—“patient-assisted teledermatology,” or “home-based teledermatology”—has been vigorously developing (7). After the first visit and face-to-face diagnosis by the dermatologist, a patient is then supposed to send pictures documenting the skin's condition. This form combines SAF teledermatology and mobile technology tools. It is particularly useful for patients with chronic diseases, such as psoriasis, atopic dermatitis, vitiligo, or leg ulcers.

One of the emerging technologies that may find application in teledermatology and implement WHO assumptions seems to be high-frequency ultrasonography (HF-USG). Although initially HF-USG was introduced to measure skin thickness, currently it has gained widespread acceptance in dermato-oncology, particularly to determine margins of skin tumors (8–10). Moreover, its application in different dermatology fields is known, particularly as a rapidly evolving method in the objective evaluation of the severity of the various chronic skin diseases (11–13). The possibility of sending images for consultation may facilitate therapeutic decisions as HF-USG can be used in a SAF manner.

We present the actual applications of 20-MHz ultrasonography in dermatology and analyze the possibility of its use as a valuable tool in teledermatology, especially in managing patients suffering from chronic skin diseases.

## Basic Principles of HF-USG

The pioneering use of the ultrasound beam in dermatology was carried out in the 1980s by Alexander and Miller with a 15-MHz probe (8). As is known, routine imaging of the abdominal organs is possible with the use of ultrasound waves in the 3–5-MHz range, while for the assessment of more superficial structures (such as lymph nodes, testes, or the thyroid gland), frequencies in the range 7.5–15 MHz are needed. The imaging of the skin became possible with the invention of transducers emitting waves with higher vibration ranges, and now ultrasound scanners of 20 MHz (High Frequency Ultrasonography, HF-USG) and higher (Ultra High Frequency Ultrasonography, UHF-USG) are used for skin examination (14). According to the Guidelines for Performing Dermatologic Ultrasound Examinations, the minimum frequency for dermatological analysis should be 15 MHz (15). However, considering the multifrequency property of available probes, the use of the range from 15 to 22 MHz for better visualization of deeper lesions is suggested (15).

The use of 20-MHz heads allows for images of structures at a depth of 8–10 mm and, according to some manufacturers, even up to 15 mm (14, 16). The higher the frequency, the better the picture resolution. The axial resolution is defined as the minimum distance that can be differentiated between two reflectors located parallel to the ultrasound beam's direction. In comparison, the lateral one is the minimum distance that can be distinguished between two reflectors situated perpendicular to the ultrasound beam's path. Fifty MHz can achieve 20 and 100 μm resolution, respectively, while 20 MHz can achieve 80 and 200 μm (17). The emission of an ultrasound beam by a piezoelectric transducer and its reception enables the analysis of echoes in various presentations. The oldest presentation is of the A-type—it analyzes the echo amplitude as a function of time and allows measurements to be made. However, most often, the B-type presentation is used to analyze the ultrasound image, which is an overlay of many A-type presentations in real time, in which echoes have been converted into glowing spots and mapped on the screen using a grayscale (256-degree grayscale) (18, 19). Thanks to the B-type presentation, it is possible to analyze the echogenicity. The echogenic structures appear bright on ultrasound images (the higher the amplitude of the reflected wave, the brighter the pixel) (14, 16, 18).

**Figure 1:**
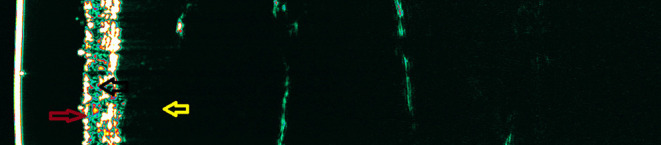
The HF-USG image of healthy skin of the forearm. Red arrow—the echo from the surface of the epidermis; black arrow—dermis; yellow arrow—subcutaneous tissue.

The ultrasound image of healthy skin corresponds to the skin layers visible on histological examination. We can distinguish three layers that differ in echogenicity (14, 18, 19) (Figure 1). The first layer, usually called entrance echo, is hyperechoic and reflects the upper (mainly dead) parts of the epidermis. Below, there is a layer of less echogenicity—which corresponds to the lower layers of the epidermis, the dermoepidermal border, and, above all, the dermis, within which echoes of varying intensity are visible. The lowest layer can be easily distinguishable because it is hypoechogenic in nature and corresponds to the subcutaneous tissue, which (depending on the anatomical location and sex) can be visualized to various depths (14, 18, 19).

## Diagnostic Properties of HF-USG In Teledermatology

According to an interesting report by Warshaw et al. (20), performed for The American Veterans Health Services Research, in-person dermatology diagnostic accuracy is of a higher quality than teledermatology. While overall management accuracy rates seem to be equivalent, in the case of malignant and premalignant lesions, rates for teledermatology and teledermatoscopy are inferior to the usual care. It is then recommended to be cautious when using teledermatology in such cases. These observations have to be considered in regard to implementing HF-USG in teledermatology modalities, particularly concerning pigmented lesions and tumors.

**Figure 2:**
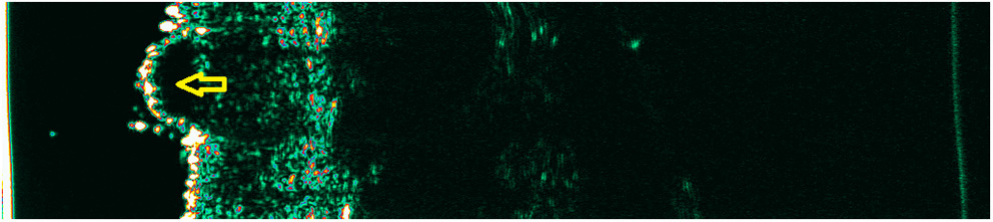
Cellular nevus. Yellow arrow—hypoechogenic mass corresponding to the nevus.

**Figure 3:**
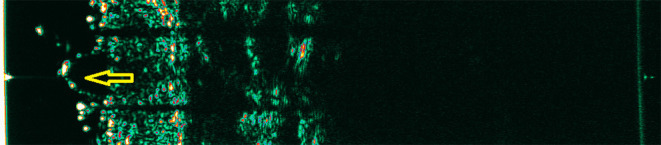
Melanoma. Yellow arrow—hypoechogenic mass corresponding to the melanoma.

Various skin pathologies change the echogenicity of its individual layers, which can be used to analyze the ultrasound image. However, the lack of sufficient resolution does not allow a full interpretation of the image with microscopic examination accuracy. Hypoechoic structures can be both of neoplastic and inflammatory origin (14, 21, 22). Moreover, an identical ultrasound image is observed in melanoma and a benign melanocytic nevus. Due to the insufficient resolution of HF-USG, the process cannot be distinguished at the cell level (Figures 2, 3). Although literature has tried to identify more specific features for various skin neoplasms, the final diagnosis should still be supported by histological examination. In melanoma, echolucent areas can be observed, with a shape depending on the subtype. The nodular form may have a spherical arrangement, while superficial spreading melanoma presents as an echolucent area parallel to the entry echo. However, there are no ultrasonographic features pathognomonic for melanoma (14).

What is more, the accuracy of ultrasound analysis may be increased with a Color Doppler ultrasound. This modality is recommended, especially for discriminating between vascular or non-vascular lesions and recognizing the benign or malignant nature of skin tumors (15, 23, 24). In previous studies, vascular signal use in assessing melanoma correlated with the Breslow index and metastatic potential (23). The addition of Color-Doppler in pigmented lesion analysis may have a significant prognostic value (25, 26).

**Figure 4:**
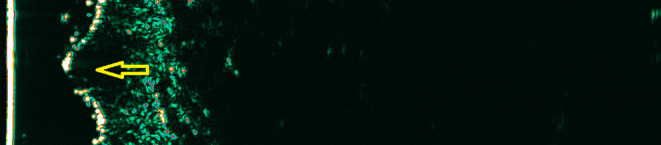
Basal cell carcinoma. Yellow arrow—hypoechogenic mass corresponding to the basal cell carcinoma.

In basal cell carcinoma (BCC), the HF-USG can detect the presence of hyperechoic spots, the so-called cotton flowers; however, in the authors' opinion, this is not a phenomenon that occurs in all tumors and therefore cannot be defining for BCC (14, 21, 27) (Figure 4). In squamous cell carcinoma (SCC) and actinic keratosis, scaling may disturb the image's interpretation. This seems to be a crucial limitation of HF-USG in evaluating hyperkeratotic tumors (10, 18, 19).

It is worth noting that the usefulness of HF-USG for assessing the thickness of BCC and melanoma infiltration has been well-documented and is now considered a recognized indication for HF-USG (14, 28–32). The preoperative determination of the surgical margins determines the future management and patients' prognosis (particularly in the case of melanoma). Previous studies confirm the agreement between a histological and ultrasonographic measurement with a high compliance rate (28–33). Therefore, the HF-USG assessment of tumor size may be then transferred to a dermato-oncologist or surgeon during teleconsultation to plan the future procedure. This way, teledermatology may globally contribute to better outcomes and radical treatment. However, it should be considered that the thickness of the tumor determined by ultrasound is slightly greater than the thickness determined histologically, due to shrinkage of the material during histological preparation and accumulation of inflammatory cells underneath the tumor (10, 24, 28). Another limitation of the proper margin assessment is the tumor size, mainly when it crosses the dermis border and penetrates the subcutaneous tissue, which is also hypoechogenic. However, the use of a multifrequency probe may reduce this limitation. The preoperative evaluation of the tumor margins may also be difficult in patients with evident photoaging, where elastosis decreases the skin echogenicity, similarly to the neoplasmatic cells (14).

Due to the low diagnostic properties of HF-USG in oncodermatology, the combination of this method with a preliminary dermoscopic evaluation may be extremely useful. Therefore, an initial diagnosis of probable pathology should be performed using a dermoscope, followed by an evaluation of disease extent using HF-USG. Acquired data, provided in the form of telecommunication, may significantly improve the diagnostic and therapeutic process. These observations remain consistent with teledermatology's role in the process of triaging (particularly the SAF method), which influences the cost-effectiveness of therapies and may limit waiting time in the case of urgent patients (34). Moreover, the inclusion of dermatoscopic images improves triaging decisions, including a need for an excision for both melanomas and other skin cancers (35).

## The Monitoring Properties of HF-USG In Teledermatology

**Figure 5:**
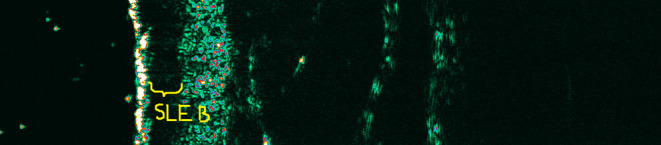
Subepidermal low echogenic band (SLEB) within the plaque of mycosis fungoides before treatment (the hypoechogenic band corresponding to the SLEB was marked).

**Figure 6:**
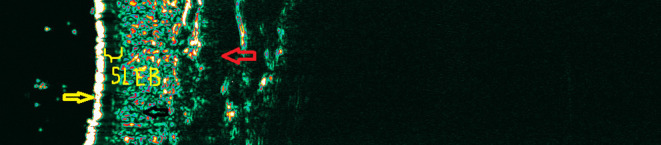
The thinning of the SLEB after treatment. Lack of complete disappearance of SLEB still shows the presence of neoplastic T cell infiltrations (the hypoechogenic band corresponding to the SLEB was marked).

Due to the limited diagnostic value of ultrasonography, the monitoring properties of HF-USG are noteworthy. They allow for observing changes dynamically—occurring in the skin over time, mainly due to the introduced treatment (Figures 5, 6). Inflammatory and neoplastic cells cause a reduction of skin echogenicity and may result in the formation of a linear band underneath the entry echo, known as the subepidermal low echogenic band (SLEB) (11, 12, 14). Originally, SLEB was observed in photodamaged skin due to glycosaminoglycan accumulation, which possesses an increased water-binding capacity (14, 18). The SLEB formation was well-documented in inflammatory diseases, including psoriasis and atopic dermatitis (11–13, 36, 37). It is not a parameter specific for any skin disease, but its changes over time are of prognostic significance, especially in patients with chronic skin diseases. The areas of reduced echogenicity are mainly related to swelling of the skin and inflammatory cell infiltration. The SLEB thickness in atopic dermatitis correlated with the degree of several histopathologic findings like epidermal hyperplasia, epidermal hyperkeratosis, the degree of parakeratosis, and the degree of spongiosis as well as the intensity of inflammatory infiltrates (38). However, in psoriatic plaque, SLEB's uniformity can be disturbed by streaky shadows perpendicular to the entry echo (most likely caused by air bubbles trapped between the scales). It is observed that SLEB thickness corresponds to the severity of skin lesions and can be used to monitor patients, also potentially as a teledermatology consultation (14, 39).

In one of our first studies, monitoring properties of HF-USG were assessed for atopic dermatitis and so-called proactive therapy, using tacrolimus ointment (40). Six-month ultrasound monitoring was performed in a group of 39 patients with atopic dermatitis (mean age 26 years). It revealed the significant decrease of SLEB value during the undergone therapy, which correlated with the disease severity assessed by measuring scales (40). We found that HF-USG allowed clinicians to visualize pathologic changes of all skin *in vivo*. As a non-invasive and independent of subjective judgment method, HF-USG should be added to the overall patient evaluation, especially in the era of evidence-based medicine (41). We presented that SLEB was detected within lesional skin and in non-affected skin regions, revealing the presence of subclinical inflammation (42, 43). Such patients may significantly benefit most from proactive treatment (40, 42). Information on the decrease of SLEB or lack of its change could be provided in the form of a teleconference to the attending physician with information about the need to intensify the treatment process. It could also be combined with an emerging teledermatology branch—patient-assisted teledermatology (also called home-based teledermatology)—which involves the SAF method and mobile technology tools. It is regarded as especially useful for patients with chronic dermatoses (psoriasis, atopic dermatitis, vitiligo, leg ulcers) and allows the specialist to monitor the phase and the degree of skin lesions. The above method is relatively new (started in 2009) and is used for both diagnosis and follow-up (20, 35).

SLEB thickness objective measurement as a useful, accurate parameter for skin assessment was also performed in psoriasis (14, 36). Queille-Roussel et al. (36) evaluated an innovative aerosol foam formulation's anti-psoriatic effect and analyzed this parameter's thickness. They found a reduction of SLEB in week 4. We also assessed monitoring properties of HF-USG in a prospective, observational study, which included 58 patients diagnosed with recurrent chronic small plaque psoriasis treated with a two-compound ointment containing calcipotriol and betamethasone dipropionate or NB-UVB (311 nm) (11). The SLEB was significantly decreased in both treated groups. The lack of its decrease, similarly to atopic dermatitis, indicated no improvement in the dermatological condition or the need to change/intensify therapy (11, 14). Such information could be consulted with a dermatologist without the need to travel and access the specialist advice more easily, especially in rural areas. What is essential, a high correlation between SLEB and PASI index indicates that HF-USG quickly complements the measurement of the severity of skin lesions in psoriasis in an objective way (11).

Monitoring properties of HF-USG seem to be particularly useful in assessing the therapeutical outcomes in patients with mycosis fungoides. They can be used in teleconsultations also with a non-dermatologist (i.e., hematologist). Neoplastic cells are responsible for decreasing echogenicity, which in mycosis fungoides also manifests in the form of SLEB, similarly to inflammatory diseases (12, 14, 44–46). We have found strong correlations between SLEB thickness and the thickness of subepidermal infiltration in histopathological examination (47). As it is known, in mycosis fungoides, the proper staging is essential in planning the therapy. It should include a detailed evaluation of the skin lesion types and an assessment of the pathological process' extent (45, 47). For 5 years, we followed up patients based on HF-USG, where SLEB was the assessed parameter (48). We evaluated well-known forms of therapies in mycosis fungoides (the effectiveness of UVA1 and PUVA). We monitored these patients' clinical responses compared to the modified Severity Weighted Assessment Tool (mSWAT) (12). Complete response in examined patients correlated with the entire disappearance of SLEB (12, 48). SLEB thickness was associated with disease severity and was wider in stage IIA patients than in stage IA and IB patients (48). For the first time, we revealed that HF-USG is a tool for evaluating the patient's response and can be applied in routine clinical practice.

What is more, using HF-USG, there is a possibility to distinguish a lesion of the type of post-inflammatory hyper/hypopigmentation from a non-specific infiltrative lesion (12, 47, 48). Performing ultrasound measurements enables the patient to be consulted with a dermatologist/hematologist without a face-to-face visit and can significantly improve the therapeutic process. In the case of irradiated patients, a routine practice in our clinic is the ultrasound supervision of patients and making decisions about additional radiation if a visible SLEB is still observed. Again, collected data can be then sent to a photodermatology center.

The practical application of HF-USG is its use in hidradenitis suppurativa (49). Recent studies proposed this method to optimize staging, treatment planning, and monitoring patients suffering from this chronic disease (49). Wortsman et al. (50) proposed a three-point sonographic scoring system for hidradenitis suppurativa, based on the number and distribution of fluid collections, fistulous tracts, and pseudocystic nodules, widening of the hair follicles, and alterations in the dermal thickness/echogenicity. According to the authors, the proper staging of these patients resulted in a management modification in 82% of cases (51). It seems that HF-USG in relation to hidradenitis suppurativa may also be conducted in the form of teleconsultation with other specialists (diagnostic and radiological center, dermatological surgeons). The possibility of an ultrasound consultation can improve diagnosis and help choose an appropriate therapeutic option (depending on the severity of skin lesions).

## The Advantages and Limitations of HF-USG

The most significant advantages of HF-USG in a monitoring examination are non-invasiveness, safety, and the possibility of multiple scans without the need to prepare the patient in advance. It seems that in the era of scrupulously determined scales determining the extent and severity of skin lesions during various treatments of skin diseases, especially to qualify patients for biological therapies and research programs, HF-USG can find an application. In the case of teledermatology, the possibility of transmitting images in between clinicians and of archiving images and analyzing them over time contributes an extremely useful visual *in vivo* method. Teleconsultation with the use of HF-USG may improve the treatment process of chronic dermatological diseases requiring long-term care. The possibility of simultaneous dermoscopy and ultrasound of the skin may have a prognostic significance in skin tumors.

Among the main limitations of HF-USG, its high price should be mentioned, as well as restricted availability of the apparatus in a doctor's office. As in the case of dermoscopy, acquiring the ability to interpret an ultrasonographic image requires appropriate training. Lately, also other techniques of non-invasive skin visualization are gaining interest in regard to associations with teledermatology—for example, reflectance confocal microscopy (RCM). RCM allows an *in vivo* evaluation of various skin lesions on the cellular level—nearly to histologic resolution. In comparison to this technique, it has to be emphasized that HF-USG does not possess sufficient resolution and its diagnostic properties are much lower (52).

## Author Contributions

All authors listed have made a substantial, direct and intellectual contribution to the work, and approved it for publication.

## Conflict of Interest

The authors declare that the research was conducted in the absence of any commercial or financial relationships that could be construed as a potential conflict of interest.
